# The CHALLENGE trial: the effects of a virtual reality-assisted exposure therapy for persistent auditory hallucinations versus supportive counselling in people with psychosis: study protocol for a randomised clinical trial

**DOI:** 10.1186/s13063-022-06683-1

**Published:** 2022-09-13

**Authors:** L. C. Smith, L. Mariegaard, D. L. Vernal, A. G. Christensen, N. Albert, N. Thomas, C. Hjorthøj, L. B. Glenthøj, M. Nordentoft

**Affiliations:** 1 Research Unit (CORE), Mental Health Center Copenhagen, Copenhagen University Hospital, Copenhagen, Capital Region of Denmark Denmark; 2 Psychiatry, Aalborg University Hospital, Aalborg, North Denmark Region Denmark; 3 Mental Health Center Esbjerg, Esbjerg, Region of South Denmark Denmark; 4 Department of Psychological Sciences, Swinburne University of Technology, Melbourne, Australia; 5 Department of Public Health, University of Copenhagen, Copenhagen, Denmark; 6 Department of Psychology, University of Copenhagen, Copenhagen, Denmark

**Keywords:** Auditory hallucinations, Voices, Avatar, Psychosis, Psychotherapy, Virtual reality

## Abstract

**Background:**

Many patients suffering from schizophrenia spectrum disorders continue having distressing auditory hallucinations in spite of treatment with antipsychotic medication. The aim of this trial is to examine the effect of a targeted virtual reality therapy for persistent auditory hallucinations in individuals with psychosis. The trial explores whether this type of therapy can decrease the severity, frequency and distress of auditory hallucinations and, additionally, whether it can reduce clinical symptoms and enhance daily functioning in individuals with psychosis.

**Methods:**

The study is a randomised, assessor-blinded parallel-group superiority clinical trial, allocating a total of 266 patients to either the experimental intervention or supportive counselling. The participants will be randomised to either (1) seven sessions of virtual reality therapy or (2) seven sessions of supportive counselling to be delivered within the first 12 weeks after inclusion in the study. All participants will be assessed at baseline and 12 and 24 weeks post-baseline. Independent assessors blinded to the treatment allocation will evaluate the outcome. The primary outcome is the level of auditory hallucinations measured with the Psychotic Symptoms Rating Scales (PSYRATS-AH) total score at the cessation of treatment at 12 weeks. Secondary outcomes are frequency of auditory hallucinations, the distress caused by auditory hallucinations, perceived voice power, patient acceptance of voices, patients’ ability to respond to voices in an assertive way and social and daily function.

**Discussion:**

Promising evidence of the efficacy of this immersive virtual reality-based therapy for auditory hallucinations exist, but evidence needs to be established in a large, methodological rigorous trial. If the therapy proves to be beneficial in reducing the severity of refractory auditory hallucinations, a large group of patients with schizophrenia and related disorders could be the target group of this short-term psychotherapeutic intervention.

## Administrative information

Note: The numbers in curly brackets in this protocol refer to the SPIRIT checklist item numbers. The order of the items has been modified to group similar items (see http://www.equator-network.org/reporting-guidelines/spirit-2013-statement-defining-standard-protocol-items-for-clinical-trials/).

**Table:** 

**Title {1}**	The CHALLENGE trial: the effects of a virtual reality-assisted exposure therapy for persistent auditory hallucinations versus supportive counselling in people with psychosis: study protocol for a randomised clinical trial
**Trial registration {2a and 2b}**	ClinicalTrials.gov NCT04661163. Date of registration: 10 December 2020.
**Protocol version {3}**	2.0
**Funding {4}**	The Innovation Fund Denmark (Innovationsfonden); Project number: IFD: 8088-00023B
**Author details {5a}**	1: Mental Health Center Copenhagen, Research Unit (CORE), Copenhagen University Hospital, Capitol Region (DK). 2: Psychiatry, Aalborg University Hospital, North Denmark Region (DK). 3: Mental Health Center Esbjerg, Region South (DK). 4: Swinburne University of Technology, Department of Psychological Sciences (AU). 5: University of Copenhagen, Department of Public Health (DK). 6: University of Copenhagen, Department of Psychology (DK).
**Name and contact information for the trial sponsor {5b}**	The Innovation Fund Denmark, info@techfunding.eu; (+45) 61 60 75 80
**Role of sponsor {5c}**	The sponsor played no part in the study design; collection, management, analysis and interpretation of the data; writing of the report; and decision to submit the report for publication.

## Introduction

### Background and rationale {6a}

Schizophrenia and related disorders tend to have an onset in late adolescence or early adulthood, with a peak incidence around 22 years of age [1] with most patients experiencing some level of symptoms for most of their life. These disorders exert tremendous suffering on both patients and relatives whilst also representing a significant burden on the larger society [2]. A substantial group of patients with first-episode psychosis achieves remission of psychotic symptoms during the first year after initial contact with mental health services. Still, almost one-third continue having psychotic symptoms in spite of treatment with antipsychotic medication [3–5]. Severe psychotic symptoms such as persistent hallucinations can have profound consequences on the patients’ ability to interact with other people. Due to hallucinations, patients may struggle to conduct daily activities such as leaving the home or using public transportation [6]. Auditory hallucinations are reported in approximately 75% of patients suffering from schizophrenia [7] and frequently appear in the form of voices with verbal content being critical of the person having the psychotic experience. Furthermore, voices may command the patient to commit aggressive, injurious or self-injurious acts [7]. Patients tend to report having only little (if any) control over the experience of hearing voices [8]. Voices are often attributed to identities speaking with purpose and with the capacity to do harm [9]. Since auditory hallucinations commonly cause high levels of distress, they have consequently become a major target of psychological therapies for psychosis [10].

### Psychotherapeutic interventions targeting auditory hallucinations

Evidence of associations between voice appraisals and distress have been reported, but review findings indicate that even though cognitive behavioural interventions are found to be successful in reappraising patients’ beliefs about their voices, they have shown relatively modest effect sizes (0.31–0.49) [11]. In order to produce greater changes, new methods may be needed. There is some emerging evidence that using experiential exercises to recreate hallucinatory episodes within a session may facilitate therapy. For example, in-session role plays [12, 13] or audio recordings of things the voice often says [14, 15] may be utilised to practise alternative responses. Using in-session exercises may be valuable in helping the person to apply what is discussed in therapy whilst the therapist can support them, aiding transfer to the self-management of hallucinations when encountered in daily life. They also offer opportunities for therapist-supported exposure to a distressing stimulus that the person may respond to in ways that exacerbate the experience, such as engaging in hostile dialogue, or that prevent habituation, such as attempting to avoid triggering or escalating the voices [16–19].

To support being able to recreate hallucinations experientially within a session, there may be particular value in the use of virtual reality. Virtual reality technology encompasses the possibility of creating artificial experiences in real time, hereby allowing the user to feel immersed and able to interact as if it were the real world, whilst within a controlled environment. A substantial number of studies have established the safety of using virtual reality in people experiencing psychosis [20].

Applied to auditory hallucinations, which are typically experienced and responded to as if sentient others are interacting with the person [21, 22], this technology offers the additional potential for voices to be represented within a virtual environment in the form of computer-generated avatars. The virtual reality exposure also allows possibilities for varying the level of difficulty of the exposure, e.g. patients can be exposed to more hostile or friendly comments. Therefore, virtual reality exposure may improve the effectiveness of the current treatment of hallucinations.

### Trials investigating virtual reality-assisted therapy targeting auditory hallucinations

To date, a small number of trials have investigated the effect of a version of virtual reality-assisted psychotherapy targeting persistent auditory hallucinations [23–25]. Following an initial proof of concept trial by Leff et al. [26], the AVATAR Therapy Trial [23] tested an intervention in which patients created a visual representation (an avatar) of the source of their auditory hallucination, presented on a computer screen in sessions. A voice transformer allowed for the therapist to talk with the tone and pitch of the distressing auditory hallucination, allowing the animated audio-visual avatar to be used in therapeutic role plays. The results from this trial revealed that 12 weeks of psychotherapy was superior to supportive counselling in reducing the severity of persistent auditory, verbal hallucinations, demonstrating a large effect size (Cohen’s *d* = 0.8). Additionally, the study showed reductions in the frequency and distress of auditory hallucinations along with a reduction in the perceived omnipotence of voices at the cessation of treatment (12 weeks). Furthermore, the therapy was well-tolerated, and no adverse events were reported [23].

In a pilot trial by du Sert et al., a similar type of psychotherapy was tested using an immersive 3D virtual reality (VR) format [24]. Findings from this study revealed significant improvements in the severity of auditory hallucinations, as well as depressive symptoms and quality of life in patients receiving VR-assisted psychotherapy (VRT) compared to treatment as usual (TAU). These beneficial effects were maintained at a 3-month follow-up [24]. The study was, however, a pilot study with only 15 participants and only reported findings on restricted outcomes. From this group of researchers, another pilot has since been conducted comparing nine sessions of VRT to nine sessions of cognitive-behavioural therapy for psychosis (CBTp) [25]. A larger effect size was found in the VRT group (Cohen’s *d* = 1.080) than in the CBTp group (Cohen’s *d* = 0.056), but the difference was not statistically significant, possibly because of the small sample size (*n* = 37 in each group). The effect was found to be maintained at a 1-year follow-up.

In a Cochrane Review of 2020 [27], it is stated that this new type of psychotherapy is still to be considered an experimental treatment and that reported studies are at risk of bias. The first of the above-mentioned studies [23, 26] were conducted by inventors of this new treatment approach, and in the latter pilot trials [24, 25], assessors were not blind to the treatment arm.

Overall, several studies provide initial positive findings of VR-assisted psychotherapy to reduce the severity of auditory hallucinations, but there is a need for replication of the findings to consolidate the reported beneficial effect in large, methodological rigours randomised clinical trial.

### Objectives {7}

The aim of this trial is to examine the effect of a targeted virtual reality-assisted therapy (VRT), for persistent auditory hallucinations in individuals with psychosis compared to the effect of supportive counselling. We want to explore whether VRT can decrease the severity, frequency and distress of auditory hallucinations and, additionally, if the VRT can reduce clinical symptoms and enhance daily functioning in individuals with psychosis.

We hypothesise that:

- Virtual reality-assisted psychotherapy will be superior to supportive counselling in reducing the primary outcome of the overall severity of auditory hallucinations in patients with psychosis.
- Virtual reality-assisted psychotherapy will be superior to supportive counselling in reducing secondary outcomes of hallucination frequency and hallucination-related distress and in improving clinical symptoms and psychosocial functioning in patients with psychosis.

Additionally, we will examine the voice-related variables perceived voice power, assertiveness in relation to voices and autonomous action in response to voices, hypothesising that each of these will show a greater change in the virtual reality assisted psychotherapy group and that change in these variables will mediate the primary outcome and changes in voice-related distress.

### Trial design {8}

The CHALLENGE trial is a multi-centre randomised, assessor-blinded parallel-group superiority clinical trial, allocating a total of 266 patients to either the experimental intervention or supportive counselling (see the “Participant timeline {13}” section) The participants will be randomised to either seven sessions of VRT or seven sessions of supportive counselling within the first 12 weeks of participation in the study. Two additional sessions of either VRT or supportive counselling are delivered within the following 12 weeks (i.e. between week 12 and week 24). All participants will be assessed at baseline and 12 and 24 weeks after baseline.

A stratified block randomisation with concealed randomisation sequence will be conducted. Independent assessors blinded to the treatment will evaluate the outcome.

## Methods: participants, interventions and outcomes

### Study setting {9}

The study setting is an outpatient routine care setting for patients suffering from schizophrenia spectrum disorders in Denmark.

### Eligibility criteria {10}

The inclusion criteria are as follows: (1) age ≥ 18 years, (2) primary diagnosis of a schizophrenia spectrum disorder (ICD-10 code: F20–F29; excluding F20.6 schizophrenia simplex and F21 schizotypal disorder as hallucinations are not contained in these disorders), (3) auditory hallucinations for at least 3 months (corresponding to a SAPS score on auditory hallucinations of ≥ 3), (4) the patient’s treatment is governed by a psychiatric outpatient facility in one of the regions conducting this study (the Capital Region, Northern or Southern Region of Denmark), (5) the patient is willing and able to give informed consent and (6) no changes in antipsychotic medication within the past 4 weeks and insufficient response to current antipsychotic compound (given the SAPS score of ≥ 3 and the fact that patients seek psychotherapy for auditory hallucinations, most referred patients cannot be said to have had sufficient effect of medication), or the patient is no longer in antipsychotic treatment but has in the past tried at least two antipsychotic compounds without sufficient treatment response.

The exclusion criteria are as follows: (1) the patient is unable to identify a single dominant voice to work with, (2) a diagnosis of organic brain disease, (3) substance abuse when leading to non-attendance at treatment or being intoxicated in sessions, (4) auditory hallucinations in a language not spoken by the therapists, (5) a command of spoken Danish or English inadequate for engaging in therapy, (6) inability to tolerate the assessment process and (7) strongly impaired vision.

### Who will take informed consent {26a}

Referred patients will undergo a process of informed consent which includes receiving written information regarding the type of intervention, potential risks/side effects and the study design. Afterwards, the patient has an appointment with one of the researchers in the CHALLENGE group who ensures that the patient understands the information given and accepts being randomly assigned to either VRT or the control group. Patients sign a consent form if they decide to participate in the study.

### Additional consent provisions for collection and use of participant data and biological specimens {26b}

On the consent form, patients can decide to accept being contacted in the future regarding the outcome of the study. Patients who decline this option still participate in the study.

### Interventions

#### Explanation for the choice of comparators {6b}

The comparator is at least seven sessions of supportive counselling during a 12-week period. Supportive counselling is the most common type of intervention already offered by mental health services in Denmark. In order to determine the efficacy of the experimental intervention (VRT), it needs to be established that it is superior to the current standard treatment offered for auditory hallucinations. The comparator cannot be said to be treatment as usual (TAU) though, i.e. supportive counselling with a more chronic patient group tend to be delivered less frequently in standard care. With the aim of comparing VRT to supportive counselling, the number of supportive counselling sessions delivered as part of the CHALLENGE trial is increased by clinicians in charge of patients’ standard care meeting a minimum of seven sessions during the first 12 weeks; some patients might receive more sessions.

#### Intervention description {11a}

*Supportive counselling (comparison group)* is managed by the psychiatric outpatient facility that governs the participant’s treatment. A minimum of seven sessions are delivered encompassing sessions with health professionals providing different types of supportive counselling, i.e. appointments with a nurse, psychiatrist, psychologist, social worker, or different types of interventions, e.g. psychotherapy or group interventions. The counselling offered is expected to be better than standard care (TAU) which often consists of fewer sessions. The type of supportive counselling can be expected to differ from one psychiatric outpatient facility to another which is also the case for patients in the experimental group who receives VRT as an add-on to TAU, in which supportive counselling can still be part of patients’ standard care.

The caseload of clinicians in outpatient facilities will vary—which is also true for clinicians in the experimental condition, since most psychotherapists in the study are primarily employed by outpatient facilities, i.e. they are working part-time in the CHALLENGE trial.

The number and type of interventions received by each patient in both outpatient facilities and (when relevant) inpatient facilities will be registered when participants have terminated their participation in the study enabling conducting of statistical analyses examining the potential between-group differences regarding mental health contacts.

**Fig. 1:**
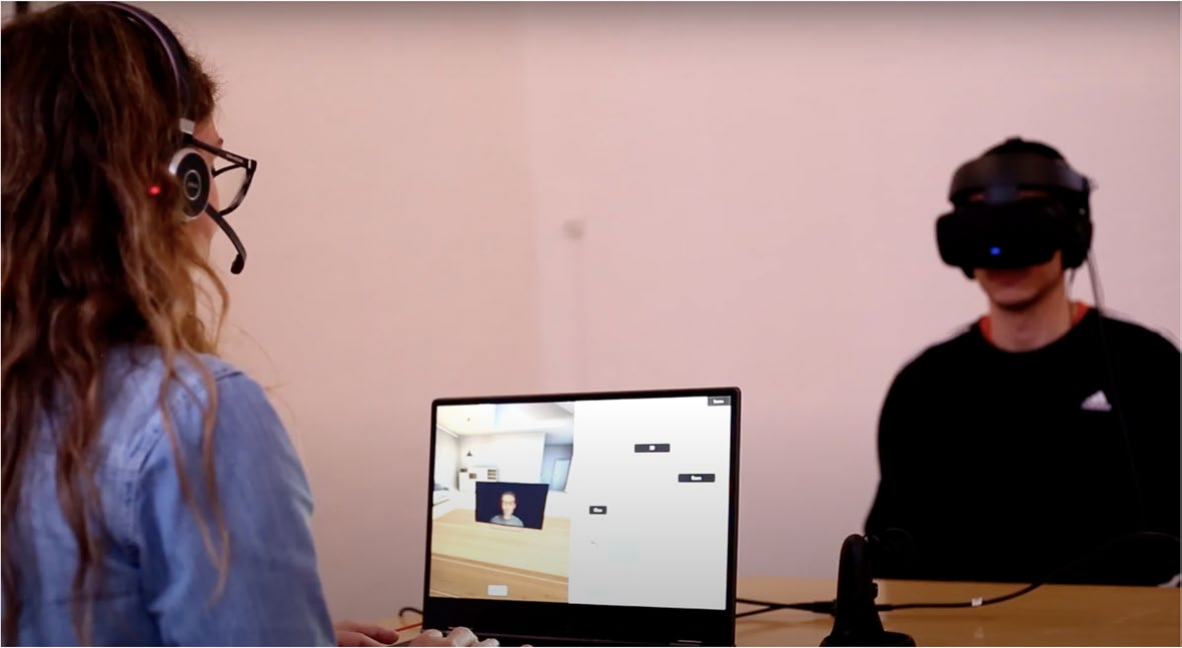
During avatar dialogues, the participant wears an Oculus Rift VR headset and noise-cancelling headphones to create an immersive experience of engaging with the representation of their voice

Patients in *the experimental treatment condition* will be offered seven individual sessions of VRT [23] within the first 12 weeks of the study and two booster sessions in the following 12 weeks, all conducted by a skilled therapist (psychologists, doctors or nurses with experience in treating patients suffering from psychosis) (Fig. 1). The therapy rests upon the exposure therapy developed by Leff et al. [23, 26]. The intervention manual of the current trial adds elements of cognitive-behavioural therapy and compassion-focused therapy. As mentioned in the previous section, most therapists are clinicians working in different psychiatric outpatient facilities (employed part-time in the CHALLENGE trial), i.e. their caseload will vary accordingly.

In the initial phase of treatment, the participants create a visual representation of their voice (either by their imagination or, for some patients, the visual hallucination corresponding to their voice). Additionally, by the use of the software program, the voice of the therapist is transformed to match the pitch and tone of the voice heard by the participant. Thereby, the voice of the computerised avatar is matched to the patients’ current auditory hallucinations. During the initial sessions, the participant is encouraged to remember important statements from their voice to form a list of verbatim statements that can be used in VR exposure therapy.

In the following sessions, the therapist initiates, encourages and supports a dialogue between the patient and the avatar by alternating between talking as the avatar, using statements from the list, and as a supportive therapist. The patient is encouraged to confront the avatar with the aim of setting about a change in the power dynamics of the relationship. During the course of therapy, the avatar will gradually change into becoming more compassionate towards the patient. Another focus of the VRT is to enhance the patient’s self-esteem. The latter is achieved partly by introducing into therapy positive statements about the patient (offered by relatives when possible) and allowing the patient to discuss these statements with the avatar.

The therapy sessions last approximately 50 min of which around 5–15 min is spent in dialogue with the avatar. The remaining time is spent on preparing the patient for the confrontation with the avatar, reflecting on the interactions with the avatar and using general cognitive behavioural techniques to reduce auditory hallucinations and associated distress.

**Fig. 2:**
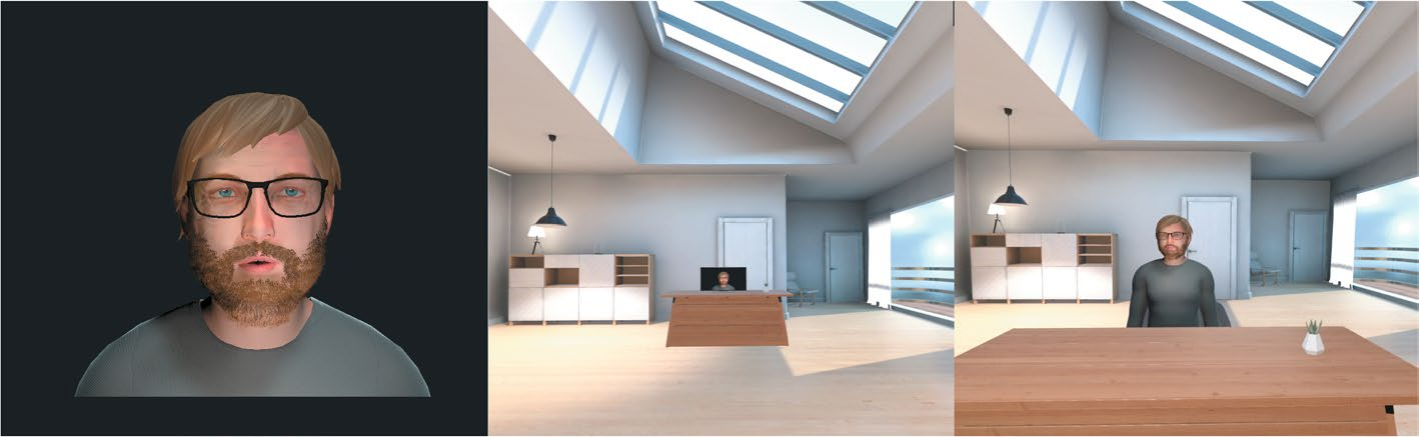
The software used for delivering the VR part of the therapy, developed by Khora VR, enables the person to interact with the avatar at different distances, allowing the therapist and patient to graduate the proximity to the avatar as needed

During avatar dialogues, the participant wears an Oculus Rift VR headset and noise-cancelling headphones to create an immersive experience of engaging with the representation of their voice (see Fig. 1). The software used for delivering the VR part of therapy was developed by Khora VR and enables the person to interact with the avatar at different distances, allowing the therapist and patient to graduate the proximity to the avatar as needed (see Fig. 2). The participant is encouraged to take a photo of the avatar and is provided with an audio recording of the dialogue with the avatar and is encouraged to listen to the recording at home to boost habituation to the exposure, to further enhance the patients’ sense of power in the relationship and to allow them to share their experience with family, friends or their regular treatment provider.

All therapists meet fortnightly with therapy lead LM for peer supervision and are supervised monthly by NT. Therapy sessions are audio-recorded and a random selection of sessions are rated for *treatment fidelity* by independent raters.

Trial participants will receive their usual medication prescribed by the psychiatrist managing their treatment. As the experimental intervention is an add-on to TAU, participants continue treatment at their regular psychiatric outpatient facility whilst receiving the VRT.

#### Criteria for discontinuing or modifying allocated interventions {11b}

Participants can choose to discontinue treatment at any time. If patients experience deterioration in their mental health status and/or are hospitalised during the treatment phase, the intervention can be modified accordingly, e.g. therapy can be conducted during a hospital stay (when considered relevant and safe) or the therapy can be paused. If deterioration may be a consequence of the intervention, the case is dealt with in supervision and severe cases (e.g. severe self-harm or suicide attempts) are also discussed in the project managing group, with the purpose of deciding if the intervention needs to be terminated.

#### Strategies to improve adherence to interventions {11c}

Therapists encourage participants to adhere to the intervention. If patients in the experimental group are afraid of standing up to the avatar or entering virtual reality, different strategies can be applied by the therapist with the aim of helping the patient overcome anxiety, for example, exposure to the computer screen with the avatar present can be chosen as a starting point for therapy instead of exposure to the full VR experience.

#### Relevant concomitant care permitted or prohibited during the trial {11d}

No special provisions.

#### Provisions for post-trial care {30}

All participants will revert to receiving their standard care within the Danish Health Care Services following the trial.

### Outcomes {12}

#### Primary outcome measure

The primary outcome is the level of auditory hallucinations measured with the Psychotic Symptoms Rating Scales (the subscale: PSYRATS-AH) [28] total score at the cessation of treatment at 12 weeks. The PSYRATS-AH is an interviewer-assessed measure tapping different dimensions of auditory hallucinations, e.g. frequency, duration and distress.

#### Secondary outcomes

Secondary outcome measures are rated at the cessation of treatment at 12 weeks and include two subscales on the PSYRATS-AH: PSYRATS-AH-Frequency [28] and PSYRATS-AH-Distress [28] measuring the frequency of auditory hallucinations and the distress caused by the hallucinations, respectively; also included are Voice Power Differential Scale [29] as a measure of perceived voice power (using the total score as the main measure, with assertiveness and power items also separately reported for comparability with previous trials), the Voices Acceptance and Action Scale (the VAAS-Action subscale specifically [30] measuring of how the patient reacts to his/her voices) and the Assertive Responding to Voices - the assertive subscale [31] (Approve – Voices; the patients’ ability to respond to voices in an assertive way). The Personal and Social Performance Scale (PSP) [32] is included as a secondary measure assessing patients’ social and daily functioning.

#### Exploratory outcomes

Exploratory outcome measures are rated at the cessation of treatment at 12 weeks and at 24 weeks of follow-up and includes the revised Beliefs About Voices Questionnaire [9] (BAVQ-R: Malevolence-, Benevolence-, Omnipotence- subscales [9]); the Voices Acceptance and Action Scale (VAAS) [30]: the VAAS acceptance subscale, and total score; Assertive Responding to Voices (Approve - Voices) [31] with aggressive and submissive responding subscales reported; the Brief Core Schema Scales: Beliefs about self and others [33]; General Self-Efficacy [34]; Emotion Regulation Questionnaire (ERQ) [35]; PSYRATS-DEL [28]; Scale for the Assessment of Positive Symptoms (SAPS) [36]; Scale for the Brief Negative Symptoms Scale (BNSS) [37]; Self-evaluation of Negative Symptoms (SNS) [38]; Calgary depression Scale [39]; WHO Well-being Index, WHO-5 [40]; Client Satisfaction Questionnaire (CSQ) [41]; Childhood Trauma Questionnaire (CTQ) [42]; Social Functioning Scale (SFS) [43]; The Suicidal Ideation Attributes Scale (SIDAS) [44] and Pittsburgh Sleep Quality Index (PSQI) [45]. Simulator Sickness Questionnaire (SSQ) [46] is administered in the experimental condition only.

The primary and secondary measures listed above are considered exploratory measures at 24-week follow-up.

### Participant timeline {13}

**Fig. 3:**
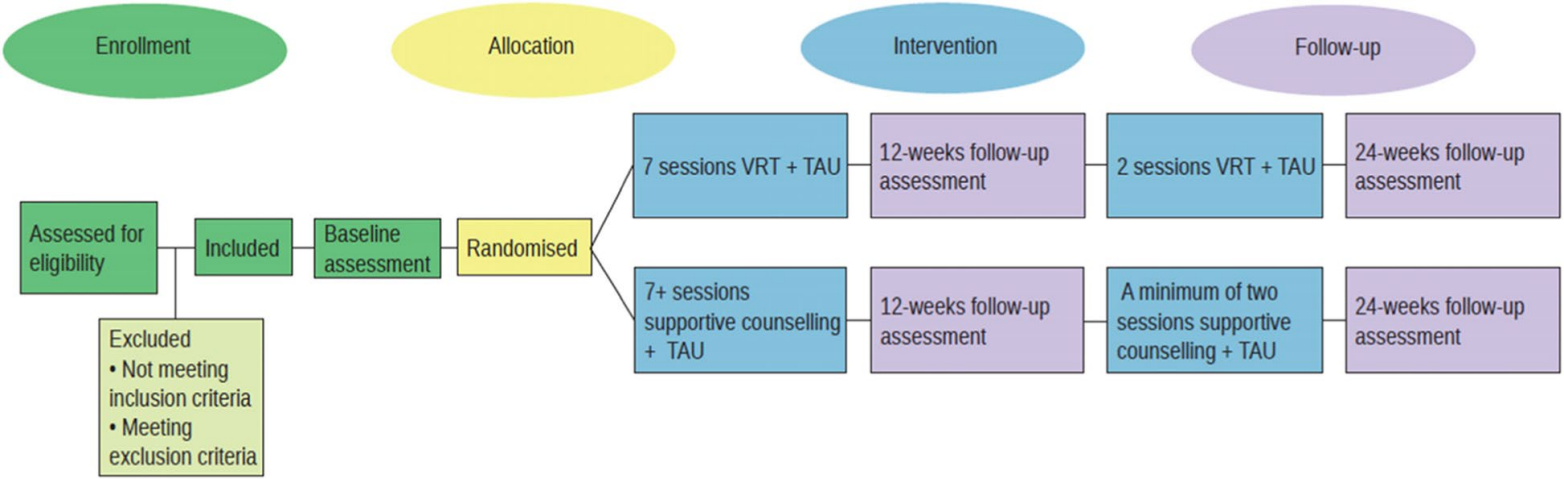
Participant timeline

The participant timeline is shown in Fig. 3.

### Sample size {14}

Sample size calculation is based on our primary outcome, a priori defined to be the between-group difference at 12 weeks on the PSYRATS-AH total score. We consider a clinically relevant between-group difference on this scale to be 3.8 (with a pooled SD of 11.0; based on calculations from the AVATAR trial [23]). With a two-sided alpha of 0.05 and a power of 80%, this will require 133 participants to be randomised to each of the two interventions (i.e. 266 participants in total).

### Recruitment {15}

The research team collaborates with clinicians from in- and outpatient facilities in the Capital Region of Denmark, the North Denmark Region, and the Region of Southern Denmark. Patients meeting the listed inclusion criteria will be approached by the clinicians who initially inform the patients about the CHALLENGE trial. When patients are interested in participation, clinicians refer them to the trial. Additionally, patients can self-refer to the trial.

### Assignment of interventions: allocation

#### Sequence generation {16a}

Patients who provide written informed consent are randomly allocated to either VRT or supportive counselling. Randomisation will be stratified by site and gender and is conducted utilising the REDCap software. REDCap is an electronic data capture tool hosted at CIMT in the Capital Region of Denmark. The randomisation programme is set up by CH (one of the authors of this article).

#### Concealment mechanism {16b}

At each site, personnel who are *not* blind to the treatment arm are responsible for the randomisation process. When a participant has been recruited for the study and baseline assessment has been conducted, the assessor informs the person responsible for conducting the randomisation process via e-mail.

Randomisation is centralised and computerised with a concealed randomisation. Block size will be unknown to the investigators and clinicians. The randomised intervention allocation is concealed until the statistical analyses of the resulting data have been completed and conclusions have been drawn.

#### Implementation {16c}

The members of the research unit of the Copenhagen Research Center for Mental Health (CORE) will be responsible for the implementation of the trial in the Capitol Region of Denmark, whilst the members of the CHALLENGE trial in the North Denmark Region (Aalborg, DK) and Region Southern Denmark (Esbjerg, DK) will be responsible for the implementation locally.

### Assignment of interventions: blinding

#### Who will be blinded {17a}

Assessors conducting the outcome evaluation are blinded. Due to the nature of the intervention, participants, therapists performing the VRT and clinicians in charge of supportive counselling or standard care are not masked.

Most assessors of the CHALLENGE trial have offices at a different location than the therapists. In one of the sites (Capital Region of Denmark), assessors’ offices are located in the same building as the therapists, but assessors and patients arriving for assessment use a different entrance to prevent unblinding.

#### Procedure for unblinding if needed {17b}

For the follow-up interviews, patients are instructed in advance not to reveal what type of treatment was received. If unblinding occurs, another assessor, blind to treatment allocation, will conduct the subsequent assessments. When possible, another assessor will perform the rating based on a video recording of the assessment (where unblinding occurred, but with this part of the video left out) in order for the patient not to have to sit through the assessment twice within a short time period.

### Data collection and management

#### Plans for assessment and collection of outcomes {18a}

Assessment will be conducted at baseline and at 12 and 24 weeks post-baseline. The PSYRATS-AH (the primary outcome of the study) has been shown to have high inter-rater reliability (ranging from 0.79 to 1.00) as well as good validity [28, 47]. Assessments will be conducted by trained psychologists at each treatment site. The main author of this article (LCS) is a psychologist with a specialisation in psychiatry. LCS is responsible for the training of all assessors. In the training process, supervised co-interviews are conducted. During the data collection phase, videotaped assessments are distributed among assessors for monthly consensus meetings. A random selection of video-taped interviews is scored individually by all assessors. ICC scores will subsequently be calculated and reported.

In case a participant meets the discontinuation or exclusion criteria during the study, or leave the intervention programme prematurely, clinical and functional assessments will still be performed, if possible.

#### Plans to promote participant retention and complete follow-up {18b}

Patients participating in the study will be contacted by telephone before the planned follow-up interviews. Assessors are flexible and can rearrange the scheduled time if needed. Patients who are in an unstable condition can be assessed at home or if hospitalised at the ward at which they are admitted. A taxi can be arranged when needed. If participants are not capable of completing the full assessment battery, the interview is abbreviated, i.e. primary and secondary outcomes are prioritised over other outcome measures.

#### Data management and confidentiality {19} {27}

Data from the patient interviews will be entered directly into the electronic case report form (CRF) using the data entry system REDCap. When necessary, the collection will be done on paper and later entered electronically. REDCap has a complete audit trail on all data transactions, detailed user rights and access control management and thereby complies with Danish legislation (Databeskyttelsesforordningen). Data for each patient is connected to a unique serial number. Only assigned researchers can access REDCap with all the data. Data on paper is stored locally and secured. Research data will be exported from REDCap without personal identifiers.

#### Plans for collection, laboratory evaluation and storage of biological specimens for genetic or molecular analysis in this trial/future uses {33}

None.

## Statistical methods

### Statistical methods for primary and secondary outcomes {20a}

Analysis of outcome will be carried out with the intention-to-treat principles. The planned comparisons between the two groups will be carried out with linear mixed model analyses with repeated measurements and an unstructured covariance matrix for normally distributed outcome measures.

No adjustments will be made for multiple comparisons, as the exploratory outcomes will primarily be used for hypothesis generation; however, interpretations regarding these exploratory analyses will be cautious for this reason.

### Interim analyses {21b}

There will be no interim analyses.

### Methods for additional analyses (e.g. subgroup analyses) {20b}

None.

### Methods in analysis to handle protocol non-adherence and any statistical methods to handle missing data {20c}

Missing data are handled implicitly by the linear mixed models through full information maximum likelihood. Analyses will be conducted according to the intention-to-treat principles, i.e. analysing individuals to their allocated groups regardless of, e.g., protocol non-adherence.

### Plans to give access to the full protocol, participant-level data and statistical code {31c}

The document constitutes the full trial protocol. Following completion of the trial, datasets and statistical code used in this study will be available from the corresponding author on reasonable requests.

### Oversight and monitoring

#### Composition of the coordinating centre and trial steering committee {5d}

The trial will be overseen by a Project Management Group (PMG) and a Trial Steering Committee (TSC). The PMG will comprise the PI of the study, the daily leader of the trial, the principal therapist of the trial and the local trial managers from each of the sites conducting the study. The PMG will have a teleconference approximately every 4–6 weeks during the recruitment period. The PMG will support any decision-making that the trial management team need further advice on.

The TSC will include representatives from (1) Mental Health Services in the Capital Region of Denmark, (2) Mental Health Services in the North and Southern Region of Denmark, (3) the Innovation Fund (the trial sponsor) and (4) Khora (the software development company involved in the study). The TSC will meet bi-annually.

#### Composition of the data monitoring committee, its role and reporting structure {21a}

There is no independent Data Monitoring Committee. The PMG will oversee trial safety and consider trial progress, especially recruitment and retention status and report to the Trial Steering Committee. The PMG can appoint additional members to assist in the process. No representatives from the trial sponsor or the IT company developing the software for the trial take part in data monitoring.

#### Adverse event reporting and harms {22}

The use of virtual reality can cause cybersickness. VR-assisted psychotherapy with patients suffering from psychosis is, however, in general considered to be well-tolerated [20]. Therefore, we do not expect any adverse events to happen. Serious adverse events will, though, be monitored and recorded throughout the study period, as well as complaints about the psychotherapy. The following are considered adverse events: (1) hospital admissions, (2) suicide attempts, (3) any violent incidents necessitating police involvement (whether victim or accused), (4) self-harming behaviour and (5) all deaths. Serious adverse events deemed related to the trial will be reported to the Committee on Health Research Ethics of the Capital Region of Denmark.

#### Frequency and plans for auditing trial conduct {23}

The PI of this study is a member of both the Trial Steering Committee and the Project Management Group thus making sure information regarding trial conduct is shared across the groups. The study will be conducted in accordance with the currently approved protocol.

#### Plans for communicating important protocol amendments to relevant parties (e.g. trial participants, ethical committees) {25}

All protocol amendments will be notified to the Committee on Health Research Ethics of the Capital Region Denmark. Deviations from the published protocol will be documented in the trial registration on ClinicalTrials.gov (NCT04661163).

#### Dissemination plans {31a}

The results of the study will be disseminated to the scientific audience, the general public and trial participants. In addition to journal publications and conference presentations, the results will be discussed with relevant patient and clinical interest groups.

## Discussion

The CHALLENGE trial is a multi-centre RCT comparing the effect of supportive counselling versus a VR-assisted psychotherapy (VRT) on the severity of auditory hallucinations in patients diagnosed with schizophrenia spectrum disorders. The trial builds on promising findings from Craig et al. [23] and du Sert et al. [24] but will have a markedly larger sample size thus becoming the largest study of its kind to date. It is a methodologically rigorous trial following the recommended guidelines aimed at reducing the risk of bias in research trials.

In the study conducted by Craig et al. [23], a significant post-treatment effect was found of the AVATAR therapy, on measures of auditive hallucinations. The statistically significant effect was, however, lost at 24 weeks of follow-up. With the aim of producing a more durable effect, the CHALLENGE trial offers two therapy sessions (booster sessions) conducted after the initial seven sessions. The hypothesis is that providing a few sessions after the initial psychotherapeutic intervention will further enhance the treatment effect and reduce the risk of relapse.

As was the case in the du Sert study (2018) [24], the psychotherapy sessions in the CHALLENGE trial are offered in a 3D VR format which has been shown to be safe and well-tolerated in patients suffering from psychosis [20]. 3D VR is considered to be of higher ecological validity [48] than a 2D virtual environment thus making possible a more immersive experience and within this more realistic environment potentially a greater treatment effect. Another potential benefit of a portable VR set-up is that it could potentially be applied in situations where the patient lives far away from the outpatient clinic or is not able to leave his/her home (e.g. due to psychiatric symptoms). Hence, the therapy could be applied to a larger target group also encompassing some of the perhaps most severe cases.

If virtual reality-assisted psychotherapy is found to be beneficial in reducing the severity of refractory auditory hallucinations, a large group of patients with schizophrenia and related disorders could potentially be the target group of the VRT. A substantial amount of the VRT sessions delivered in the CHALLENGE trial is conducted by clinicians working in psychiatric outpatient facilities, thus inherent in the trial design is a testing of the VRT’s applicability in everyday work settings. The trial may hereby inform a potential implementation of the VRT in clinical practice. The VRT under study consists of merely nine sessions further enhancing its potential implementation in psychiatric outpatient settings in which resources unfortunately tend to be limited.

### Limitations and future studies

As a consequence of the inclusion and exclusion criteria applied in the CHALLENGE trial, the patient group under study is primarily patients with treatment-resistant schizophrenia. Recruiting patients with first-episode psychosis or medicine-naïve patients could be of interest for future studies. Also, other patient groups suffering from critical or hostile voices (e.g. patients with psychotic depression) might benefit from this type of intervention.

The experimental condition of this trial was conducted with the use of 3D VR, i.e. the participant wears a VR headset and noise-cancelling headphones and is immersed in a 3D virtual environment. The use of 3D VR might turn out to be more anxiety-provoking for the population under study, and its potential benefits might come with the risk of fewer patients entering the study and higher drop-out rates. Future studies might explore if the intervention is better tolerated in 2D or 3D and whether there are differences in effect size between the two formats.

The control group of this trial is supportive counselling governed by a variety of outpatient mental health clinics. As a consequence of the trial design, we cannot control for the treatment effect of other types of interventions (e.g. medicine adjustments during the trial period, participants attending groups for voice hearers or patients engaging in other psychotherapeutic interventions which may be more likely to be delivered to the standard group than to patients within the experimental condition). It can be stated though, that the VRT under study is currently not delivered as a component of standard care and as such it can be regarded relevant to test whether the VRT appears to exhibit superiority over supportive counselling and TAU in the treatment of auditory hallucinations [49].

## Trial status

The CHALLENGE trial is currently recruiting in all three regions of Denmark (i.e. Capitol Region, North Denmark Region and the Southern Region of Denmark). The first patient was included on November 16, 2020. Recruitment is due to end in December 2022 and follow-up completed in June 2023. There have been obstacles in regard to recruitment caused by the COVID-19 pandemic and the recruitment period may be prolonged accordingly.

## Data Availability

LCS, LM, DLV, CH, LBG and MN will have full access to the final dataset.

## Abbreviations

2D: Two-dimensional
3D: Three-dimensional
CBT: Cognitive behavioural therapy
PI: Principal investigator
TAU: Treatment as usual
VR: Virtual reality
VRT: Virtual reality-assisted psychotherapy
